# Bio-Guided Isolation of the Cytotoxic Terpenoids from the Roots of *Euphorbia kansui* against Human Normal Cell Lines L-O2 and GES-1

**DOI:** 10.3390/ijms130911247

**Published:** 2012-09-10

**Authors:** Li Zhang, Lan Gao, Zhengjun Li, Xiaojing Yan, Yanjing Yang, Yuping Tang, Yudan Cao, Anwei Ding

**Affiliations:** Jiangsu Key Laboratory for High Technology Research of TCM Formulae, Nanjing University of Chinese Medicine, Nanjing 210046, China; E-Mails: zhangli@njutcm.edu.cn (L.Z.); gaolan1000@126.com (L.G.); lizhengjun404@126.com (Z.L.); yanxiaojing963@163.com (X.Y.); candy-jing@qq.com (Y.Y.); raindc@163.com (Y.C.)

**Keywords:** *Euphorbia kansui*, terpenoids, cytotoxicity, bio-guided separation, human normal liver cell lines L-O2, human normal gastric epithelial cell line GES-1

## Abstract

The dried roots of *Euphorbia kansui* (kansui) have been used for centuries in China as a herbal medicine for edema, ascites, and asthma. The 95% ethanol extract showed a significant inhibition of cell proliferation against human normal cell lines L-O2 and GES-1. Bioassay-guided separation of the 95% ethanol extract from the roots of *E. kansui* led to the isolation of 12 diverse terpenoids whose structures were identified by ^1^H, ^13^C NMR spectroscopy and ESI-MS as kansuinine A (**1**), kansuinine B (**2**), kansuinine C (**3**), kansuiphorin C (**4**), 3-*O*-(2′*E*,4′*Z*-decadienoyl)-20-*O*-acetylingenol (**5**), 3-*O*-(2′*E*,4′*E*decadienoyl)-20-*O*-acetylingenol (**6**), 3-*O*-(2′*E*,4′*Z*-decadienoyl)-20-deoxyingenol (**7**), 3-*O*-benzoyl-20-deoxyingenol (**8**), 5-*O*-benzoyl-20-deoxyingenol (**9**), kansenone (**10**), *epi*-kansenone (**11**), euphol (**12**). All these 12 terpernoids were evaluated *in vitro* for cytotoxicity on L-O2 and GES-1 cell lines. Most ingenane-type diterpenoids and 8-ene-7-one triterpenoids (**5**–**11**) exhibited a relatively lower IC_50_ value; therefore, these compounds had stronger cytotoxicity against human normal cell lines L-O2 and GES-1 with dose-dependent relationships. These results will be significantly helpful to reveal the mechanism of toxicity of kansui and to effectively guide safer clinical application of this herb.

## 1. Introduction

With the development of science and technology, and the increasing health awareness and life expectancy of humans, the safety problem of traditional Chinese medicines (TCM) is attracting more and more attention. The dried roots of *Euphorbia kansui* T.N. Liou ex T.P. Wang, known as “kansui,” which were recorded in *Shennong-Bencao* and have been used for centuries in China as a herbal medicine for edema, ascites, and asthma [1,2]. Recently, it was found that kansui has excellent prospects for the treatment of cancer [2–6], pancreatitis [7–9], and intestinal obstruction [10,11]. However, kansui causes severe skin, oral, and gastrointestinal irritation, hepatic injury and tumor promoting toxicity, which have seriously restricted its clinical application [12–16]. So, outweighing all other considerations is the need to understand the toxicity mechanism of kansui, and further to reduce its toxicity without affecting its therapeutic action.

Previous phytochemical investigation resulted in several terpenoids and phenolic derivatives isolated and identified from kansui [13,17–23]. It was reported that the terpenoids in kansui, including jatrophane-type diterpenoids, ingenane-type diterpenoids and triterpenoids, showed comprehensive bio-activities such as the anti-leukemia [2], anti-tumor [2–5], anti-allergy [24], anti-virus [13,25], and antinematodal activity [26]. On the other hand, some of the terpenoids had a serious proinflammatory effect [27,28]. Our recent study also discovered that kansui exhibited serious gastrointestinal toxicity and hepatotoxicity [14–16,29]. In the present study, the cytotoxicity of kansui extracts was evaluated *in vitro* against human normal cell lines L-O2 and GES-1. The 95% ethanol extract showed a significant inhibition of cell proliferation against two human normal cell lines. Bioassay-guided isolation of pure compounds was carried out, their structures were identified, and their structure-activity relationships were further studied.

## 2. Results and Discussion

### 2.1. Identification of Compounds 1–12

**Figure 1:**
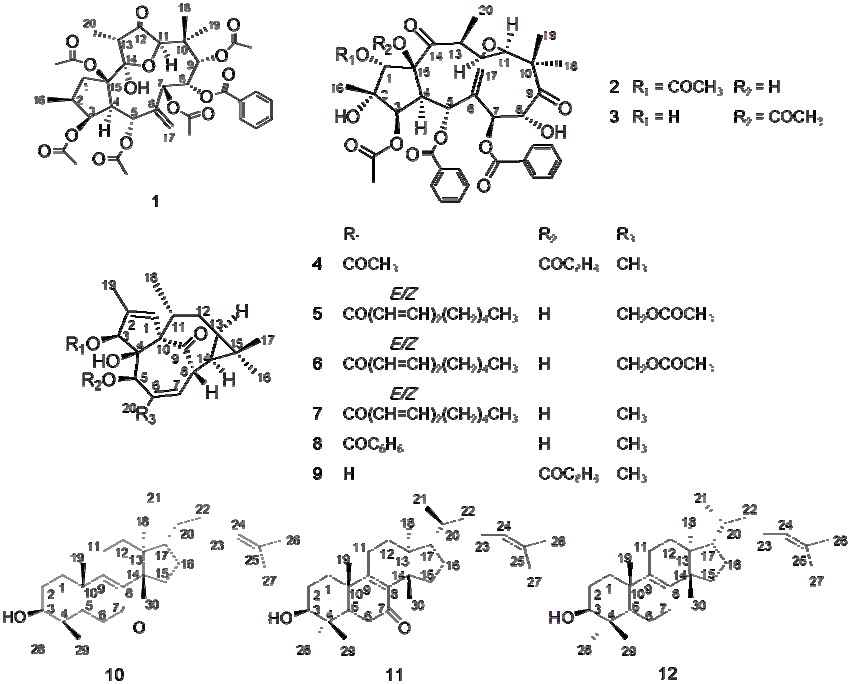
The chemical structures of compounds **1**–**12**.

On the basis of cytotoxicity assay results of different extracts and pure compounds against human normal cell lines L-O2 and GES-1 (Table 4), 12 terpenoids were isolated from the EtOAc extract of kansui. By spectral and physiochemical data analysis and/or comparison with literatures data, their structures were elucidated as kansuinines A, B, C (**1**–**3**) [17,21], kansuiphorin C (**4**) [20], 3-*O*-(2′*E*,4′*Z*decadienoyl)-20-*O*-acetylingenol (**5**) [17], 3-*O*-(2′*E*,4′*E*-decadienoyl)-20-*O*-acetylingenol (**6**) [17], 3-*O*-(2′*E*,4′*Z*-decadienoyl)-20-deoxyingenol (**7**) [17,23], 3-*O*-benzoyl-20-deoxyingenol (**8**) [22], 5-*O*-benzoyl-20-deoxyingenol (**9**) [17], kansenone (**10**) [17], *epi*-kansenone (**11**) [17], and euphol (**12**) [17,21] (Figure 1). This was the first report of the bio-guided isolation of different fractions and pure terpenoids from kansui extracts by the gastrointestinal and hepatic cytotoxicity assay against human normal cell lines L-O2 and GES-1.

Kansuinine A (**1**): white powder, mp 218–220 °C; *R*_f_ = 0.25 (SiO_2_, petroleum ether-chloroform-EtOAc = 5:3:3); UV (MeOH) *λ*_max_ 232 nm; ESI-MS (positive) *m/z* 753 [M + Na]^+; 1^H-NMR data, see Table 1.

Kansuinine B (**2**): colorless crystal; mp 215–216 °C; *R*_f_ = 0.30 (SiO_2_, petroleum ether-chloroform-EtOAc = 5:3:3); UV (MeOH) *λ*_max_ 232 nm; ESI-MS (positive) *m/z* 745 [M + Na]^+; 1^H-NMR data, see Table 1.

Kansuinine C (**3**): colorless needle crystal; mp 217–218 °C; *R*_f_ = 0.28 (SiO_2_, petroleum ether-chloroform-EtOAc = 5:3:3); UV (MeOH) *λ*_max_ 232 nm; ESI-MS (positive) *m/z* 745 [M + Na]^+; 1^H-NMR data, see Table 1.

Kansuiphorin C (**4**): colorless gum; *R*_f_ = 0.83 (SiO_2_, petroleum ether-chloroform-EtOAc = 5:3:1); UV (MeOH) *λ*_max_ 230; ESI-MS (positive) *m/z* 501 [M + Na]^+; 1^H-NMR data, see Table 2.

3-*O*-(2′*E*,4′*Z*-Decadienoyl)-20-*O*-acetylingenol (**5**): colorless gum; *R*_f_ = 0.55 (SiO_2_, petroleum ether-chloroform-EtOAc = 5:3:2); UV (MeOH) *λ*_max_ 266, 206; ESI-MS (positive) *m/z* 563 [M + Na]^+; 1^H-NMR data, see Table 2.

3-*O*-(2′*E*,4′*E*-Decadienoyl)-20-*O*-acetylingenol (**6**): colorless gum; *R*_f_ = 0.65 (SiO_2_, petroleum ether-chloroform-EtOAc = 5:3:2); UV (MeOH) *λ*_max_ 266, 203; ESI-MS (positive) *m/z* 563 [M + Na]^+; 1^H-NMR data, see Table 2.

3-*O*-(2′*E*,4′*Z*-Decadienoyl)-20-deoxyingenol (**7**): colorless gum; *R*_f_ = 0.66 (SiO_2_, petroleum ether-chloroform-EtOAc = 5:3:2); UV (MeOH) *λ*_max_ 260, 206; ESI-MS (positive) *m/z* 505 [M + Na]^+; 1^H-NMR data, see Table 2.

3-*O*-Benzoyl-20-deoxyingenol (**8**): colorless gum; *R*_f_ = 0.72 (SiO_2_, petroleum ether-chloroform-EtOAc = 5:3:2); UV (MeOH) *λ*_max_ 273, 229, 208; ESI-MS (positive) *m/z* 459 [M + Na]^+; 1^H-NMR data, see Table 2.

5-*O*-Benzoyl-20-deoxyingenol (**9**): colorless gum; *R*_f_ = 0.61 (SiO_2_, petroleum ether-chloroform-EtOAc = 5:3:2); UV (MeOH) *λ*_max_ 273, 229, 208; ESI-MS (positive) *m/z* 459 [M + Na]^+; 1^H-NMR data, see Table 2.

Kansenone (**10**): colorless gum; *R*_f_ = 0.61 (SiO_2_, petroleum ether-chloroform-EtOAc = 5:3:2); UV (MeOH) *λ*_max_ 252; ESI-MS (positive) *m/z* 441 [M+H]^+; 1^H-NMR data, see Table 3.

*Epi*-kansenone (**11**): colorless gum; *R*_f_ = 0.64 (SiO_2_, petroleum ether-chloroform-EtOAc = 5:3:2); UV (MeOH) *λ*_max_ 252; ESI-MS (positive) *m/z* 441 [M + H]^+; 1^H-NMR data, see Table 3.

Euphol (**12**): white crystal (ethyl acetate); mp 116–118 °C; *R*_f_ = 0.52 (SiO_2_, hexane-acetone-glacial acetic acid = 8:1:0.1); UV (MeOH) *λ*_max_ 200; ^1^H-NMR (300 MHz, CDCl_3_) δ 0.75 (3H, s, 30-H), 0.80 (3H, s, 18-H), 0.84 (3H, d *J* = 3.3 Hz, 21-H), 0.87 (3H, s, 28-H), 0.95 (3H, s, 19-H), 1.00 (3H, s, 29-H), 1.60 (3H, s, 27-H), 1.68 (3H, s, 26-H), 3.24 (1H, dd *J* = 4.8, 11.6 Hz, 3α-H), 5.09 (1H, t, *J* = 7.5 Hz, 24-H); ^13^C-NMR (75 MHz, CDCl_3_): 35.4 (C-1), 27.7 (C-2), 78.9 (C-3), 37.3 (C-4), 50.9 (C-5), 18.9 (C-6), 27.9 (C-7), 134.0 (C-8), 133.5 (C-9), 38.9 (C-10), 21.5 (C-11), 28.1 (C-12), 44.1 (C-13), 49.9 (C-14), 30.9 (C-15), 29.8 (C-16), 49.6 (C-17), 15.6 (C-18), 20.1 (C-19), 35.8 (C-20), 18.9 (C-21), 35.2 (C-22), 24.7 (C-23), 125.2 (C-24), 130.8 (C-25), 25.7 (C-26), 17.7 (C-27), 24.4 (C-28), 28.0 (C-29), 15.5 (C-30).

### *2.2. Cytotoxicity Activity on Human Normal Cell Lines of Extracts and Compounds from* Kansui

As shown in Table 4, the EtOH and EtOAc extract exhibited significant inhibition of cell proliferation against two human normal cell lines L-O2 and GES-1 with dose-dependent relationship. The IC_50_ values of EtOH extract on L-O2 and GES-1 were 42.02 and 30.67 μg/mL, respectively. And the cytotoxicity of EtOAc extract on L-O2 and GES-1 were stronger than that of EtOH extract. The IC_50_ values of EtOAc extract on L-O2 and GES-1 were 27.08 and 21.89 μg/mL, respectively. The water extract showed no obvious cytotoxicity against these two human normal cell lines.

**Figure 2:**
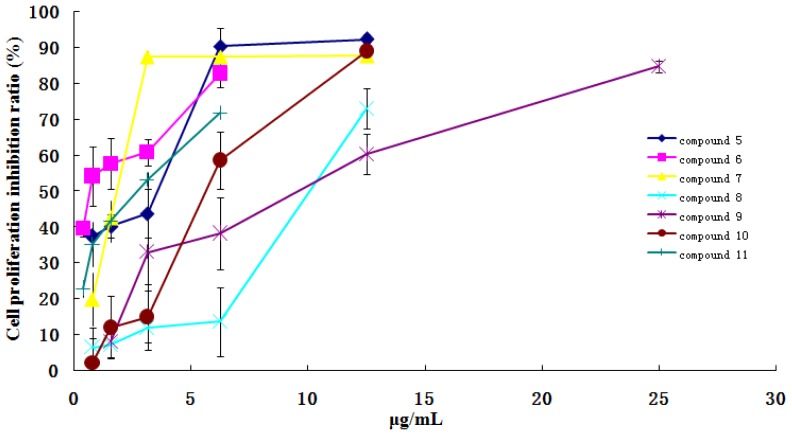
The cytotoxicity and dose-dependent relationship of compounds **5**–**11** on human normal cell lines GES-1.

**Figure 3:**
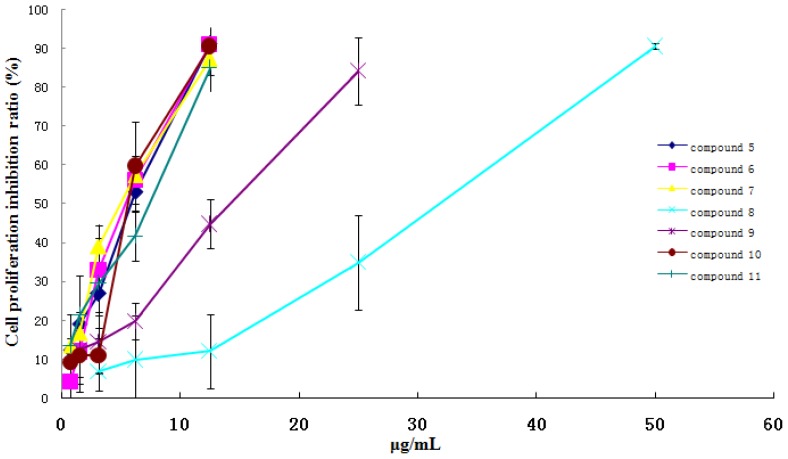
The cytotoxicity and dose-dependent relationship of compounds **5**–**11** on human normal cell lines L-O2.

Compared with the control group, compounds **5**, **7**, **8** and **10** in the concentration range of 0.78–12.5 μg/mL, compounds **6** and **11** in the concentration range of 0.39–6.25 μg/mL, and compound **9** in the concentration range of 1.56–25 μg/mL, showed significant inhibition activity on GES-1 cell lines growth with a dose-dependent relationship (Figure 2). And compounds **5**, **6**, **7**, **10** and **11** in the concentration range of 0.78–12.5 μg/mL, compound **9** in the concentration range of 1.56–25 μg/mL, and compound **8** in the concentration range of 3.125–50 μg/mL, had significant inhibition activity on L-O2 cell lines growth with a dose-dependent relationship (Figure 3). Whereas, other compounds did not show the obvious cytotoxicity activity in the concentration range of 3.125–50 μg/mL. Considering their structure, the results indicated that ingenane-type diterpenoids (**5**–**9**) except compound **4** and 8-ene-7-one triterpenoids (**10** and **11**) in kansui possessed stronger gastrointestinal toxicity and hepatotoxicity than other compounds. More interestingly, the IC_50_ values of **5**–**11** and the kansui extracts on GES-1 cell lines were less than those on L-O2 cell lines, which suggested that the gastrointestinal toxicity of kansui is stronger than its hepatotoxicity. This result coincided with the clinical toxic performance of kansui [14–16].

## 3. Experimental Section

### 3.1. Chemicals and Reagents

Analytical grade ethanol, EtOAc and petroleum ether (60–90 °C) (Sinopharm Chemical Reagent Co., Ltd. Shanghai, China) were used for extraction and isolation. The silica gel (Qingdao Ocean chemical industry, Qingdao, China) was used for column chromatography. Dulbecco’s Modified Eagle’s Medium (DMEM) was purchased from Gibco Co., Ltd. (Grand Island, NY, USA); fetal bovine serum (FBS) and calf serum (CS) were purchased from Sijiqing Biological Engineering Material Co., Ltd. (Hangzhou, China); 3-(4,5-dimethylthiazol-2-yl)-2,5-diphenyltetrazolium bromide (MTT) was obtained from Beijing Solarbio science and technology Co., Ltd. (Beijing, China); PBS buffer was purchased from Boster Bio-engineering Co., Ltd. (Wuhan, China); dimethylsulfoxide (DMSO) was purchased from Sinopharm Chemical Reagent Co., Ltd. (A·R grade, Shanghai, China).

### 3.2. Instrumentation

Melting points were determined on a Yanagimoto MP-500 D micro-melting point apparatus and are uncorrected. The UV spectrum was obtained in MeOH on a Shimadzu UV-2401 spectrophotometer. The NMR spectra were recorded on Bruker Avance AV 300 instrument operating at 300 MHz for ^1^H and 75 MHz for ^13^C, using standard pulse sequences. Chemical shifts are reported on the δ scale in parts per million, with TMS as an internal standard. The electrospray ionization mass spectra (ESI-MS) were obtained on a Q-TOF Micro mass spectrometer (Waters Company, Milford, MA, USA). Thin-layer chromatography (TLC) was performed on Qingdao Ocean TLC plates (0.25 mm thickness, Qingdao Ocean chemical industry, Qingdao, China), with compounds visualized by spraying with 10% (*v*/*v*) H_2_SO_4_ in ethanol solution and then heating on a hot plate. Semipreparative HPLC was performed on a Hanbang NP 7000 apparatus with a NU 3000 detector. A Hanbang Phecda Si silica gel column (20 × 250 mm i.d., 5 μm) was used for preparative purposes. Laminar flow clean bench (Model: SW-CJ-1F) was purchased from Suzhou Purification Equipment Co., Ltd. (Suzhou, China); CO_2_ incubator (Model: MCO-20AIC) was purchased from Sanyo Denki Co., Ltd. (Moriguchi, Osaka, Japan); Microplate Reader (model: 680) obtained from Bio-Rad Laboratories (Hercules, CA, USA). Inverted phase contrast microscope (model: CKX31) was purchased from Olympus Co., Ltd. (Tokyo, Japan).

### 3.3. Plant Material

The roots of *Euphorbia kansui* T.N. Liou ex T.P. Wang were collected from Red River valley of Baoji (Shanxi Province, China). The crude plant was identified by Professor Chungen Wang (Nanjing University of Chinese Medicine, Nanjing, China). The voucher specimens (No. NJUTCM-20091008) was deposited in the Herbarium of Nanjing University of Chinese Medicine, Jiangsu, China.

### 3.4. Extraction and Isolation

The dried and crushed roots of *E. kansui* (20.0 kg) were extracted with 95% EtOH under 50 °C water bath, the supernatant was separated every day, and the solvent was removed under reduced pressure, and the residue (2.35 kg) was partitioned with ethyl acetate and water to provide the EtOAc fraction (1.032 kg) and water fraction (1.056 kg). The EtOAc fraction 0.904 kg was subjected to silica gel column chromatography (100 × 10 cm, 200–300 mesh, eluted with petroleum ether and ethyl acetate in increasing polarity). The column chromatographic eluents (500 mL each) were concentrated under reduced pressure and combined according to TLC monitoring into 10 fractions. Fraction 1 was subjected to silica gel column chromatography eluted with petroleum ether-ethyl acetate 100:1, and was further recrystallized to give compound **12** (a large amount). Fractions 2–4 was subjected to silica gel column chromatography eluted with petroleum ether-ethyl acetate from 100:16 to 100:18, and were further recrystallized to give **1** (132 mg), **2** (86 mg) and **3** (46 mg). Fraction 5 was subjected to silica gel column chromatography eluted with petroleum ether-ethyl acetate 100:2, and was further purified by preparative TLC using silica gel (petroleum ether-chloroform-ethyl acetate 5:3:1, *R*_f_ = 0.83) to give compound **4** (106 mg). Fraction 6 was subjected to silica gel column chromatography eluted with petroleum ether-ethyl acetate 100:5, and was further purified by preparative HPLC (petroleum ether-ethyl acetate, *ρ* 0.705, UV detector set at 260 nm, flow rate at 15 mL/min) to give compound **9** (41 mg, *t*_R_ 31.0 min). Fraction 7 was subjected to silica gel column chromatography eluted with petroleum ether-ethyl acetate 100:5, and was further purified by preparative HPLC (petroleum ether-ethyl acetate, *ρ* 0.700, UV detector set at 260 nm, flow rate at 15 mL/min) to give compound **7** (56 mg, *t*_R_ 14.3 min). Fraction 8 was subjected to silica gel column chromatography eluted with petroleum ether-ethyl acetate 100:6, and was further purified by preparative HPLC (petroleum ether-ethyl acetate, *ρ* 0.692, UV detector set at 260 nm, flow rate at 15 mL/min) to give compound **8** (14 mg, *t*_R_ 16.4 min). Fraction 9 was subjected to silica gel column chromatography eluted with petroleum ether-ethyl acetate 100:8, and was further purified by preparative HPLC (petroleum ether-ethyl acetate, *ρ* 0.706, UV detector set at 260 nm, flow rate at 15 mL/min) to give compound **10** (73 mg, *t*_R_ 20.6 min) and **11** (32 mg, *t*_R_ 22.7 min). Fraction 10 was subjected to silica gel column chromatography eluted with petroleum ether-ethyl acetate 100:11, and was further purified by preparative HPLC (petroleum ether-ethyl acetate, *ρ* 0.705, UV detector set at 260 nm, flow rate at 15 mL/min) to give compound **5** (29 mg, *t*_R_ 19.0 min) and **6** (21 mg, *t*_R_ 20.3 min).

### 3.5. Cell Line and Cell Culture

Human normal liver cell L-O2 was kindly donated by the School of Life Science, Nanjing Normal University (Nanjing, China), and Human normal gastric epithelial cell GES-1 was kindly donated by the Department of Gastroenterology, Nanjing Drum Tower Hospital (Nanjing, China). Human normal liver cell LO-2 was routinely cultured in DMEM medium [16], supplemented with 10% (*v*/*v*) fetal bovine serum (FBS), 100 U/mL of penicillin and 100 μg/mL of streptomycin in a humidified atmosphere with 5% CO_2_ at 37 °C in an incubator. The culture medium was changed every day. Cultures were dissociated with 0.25% trypsase in phosphate buffered (pH 7.2~7.4) saline (PBS). Human normal gastric epithelial cell GES-1 was routinely cultured in DMEM medium [30], supplemented with 15% (*v*/*v*) calf serum (CS), 100 U/mL of penicillin and 100 μg/mL of streptomycin in a humidified atmosphere with 5% CO_2_ at 37 °C in an incubator. The culture medium was changed every two days. Cultures were dissociated with 0.25% trypsase and 0.05% EDTA in PBS (pH 7.2~7.4).

### 3.6. Cell Proliferation Analysis

Cytotoxicity activity was assayed by MTT method as described [31]. Briefly, cells were seeded in 96-well plates (Costar, Corning Co., Ltd., Beijing, China) with 100 μL at a concentration of cell suspension of 1.0 × 10_4_ cells/mL (L-O2 cell) and 1.0 × 10_5_ cells/mL (GES-1 cell), and incubated for 24 h at 37 °C in a humidified atmosphere of 5% CO_2_ in an incubator.

Every sample was solubilized in dimethyl sulfoxide (DMSO) to a concentration of 10 mg/mL and stored at 4 °C, then diluted with complete medium to the desired series concentration before treating the cells. Each solution was added into 96-well plates with 100 μL per well, repeated 6 well. Cells of the control groups were treated with the same volume of medium. The treated cells with different groups were incubated for 48 h at 37 °C in a humidified atmosphere of 5% CO_2_ in an incubator. Then, the treated cells were added with 20 μL/well of MTT (5.0 mg/mL), and incubated for a further 4 h in an incubator. The growth medium was removed from all the wells, and 150 μL DMSO were added to each well. The plates were shaken gently for 10 min to blend the mixture. The absorbance value of each well was read at 490 nm using a microplate reader. All experiments were performed at least three times. The inhibition rate of cell proliferation was calculated according to the formula:

(1) cell inhibition (%)=1-sample solution absorbance value/control absorbance value)×100%

### 3.7. Statistical Analysis

The results were expressed as an inhibition ratio and IC_50_ (concentration that inhibits 50% of cell growth). The data were analyzed with software SPSS 15.0 (SPSS Inc.: Chicago, IL, USA). The anti-proliferation activity is more significant when the IC_50_ is smaller.

## 4. Conclusions

In this paper, the 95% EtOH extract of kansui exhibited significant activity against two human normal cell lines GES-1 and L-O2. By bio-guided isolation, 12 terpenoid compounds, including jatrophane-type diterpenoids (**1**–**3**) ingenane-type diterpenoids (**4**–**9**) and triterpenoids (**10**–**12**), were isolated and identified from the active fraction of kansui. The results showed that five ingenane-type diterpenoids (**5**–**9**) and two triterpenoids (**10** and **11**) exhibited relative lower IC_50_ value and thus, stronger potential cytotoxic activity against different human normal cell lines in a dose-dependent fashion. The ingenane-type diterpenoids with 3-unsaturated aliphatic chain, such as compounds **5**, **6** and **7**, possessed stronger cytotoxic activity. And the cytotoxic activity was slightly decreased if the 3-unsaturated aliphatic chain was replaced by other groups, such as methyl and phenyl groups; and there was the same decrease of cytotoxic activity if the 5-hydroxyl group was replaced by a benzoyl group. And 20-*O* group had little influence on the cytotoxic activity. The three jatrophane-type diterpenoids (**1**–**3**) showed very weak cytotoxic activity against the two human normal cell lines. The triterpenoids with 8-ene-7-one showed stronger cytotoxic activity. For example, compound **10** (kansenone) and **11** (Epi-kansenone) had stronger cytotoxic activity than that of compound **12** (euphol). It is also notable that all identified toxic terpenoids were also reported in the literature to be important pharmaceutical ingredients for anti-tumor, anti-allergy, anti-virus, and antinematodal activity [2–5,13,17–19]. Therefore, it is essential that the dose of kansui should be controlled within an appropriate range in the clinical application to assure its safe and efficacy.

## Figures and Tables

**Table 1:** The ^1^H-NMR spectral assignments (δ/ppm) for compounds **1**–**3** in CDCl_3_ (300 MHz).

Position	1	Position	2	3
1	2.66 (dd, 6.3, 14.1)	1	4.33 (brs)	4.94 (s)
	2.25 (m)			
2	2.10 (m)	2	-	-
3	5.60 (brs)	3	5.53 (d, 4.8)	5.38 (d, 4.7)
4	2.98 (brs)	4	3.63 (dd, 11.4, 5.0)	3.48 (m)
5	6.15 (s)	5	5.93 (d, 5.85)	5.95 (s)
7	6.44 (s)	7	5.88 (s)	5.88(s)
8	6.05 (s)	8	4.66 (brs)	4.70 (d, 9.3)
9	5.09 (s)	9	-	-
11	4.14 (s)	11	3.70 (d, 1.8)	3.65 (d, 2.04)
12	-	12	3.33 (m)	3.46 (m)
13	2.33 (q, 6.6)	13	3.38 (m)	3.26 (m)
16	0.936 (d, 6.6)	16	1.29 (s)	1.32 (s)
17	5.24 (s)	17	6.52 (s)	6.390 (s)
	5.14 (s)		5.94 (s)	5.95 (s)
18	1.31 (s)	18	1.35 (s)	1.33 (s)
19	1.15 (s)	19	0.85 (s)	0.89 (s)
20	1.32 (d, 6.3)	20	1.53 (d, 6.27)	1.65 (d, 6.51)
Acetyls	-	Acetyls	-	-
COMe-1	-	COMe-1	-	2.14 (s)
COMe-3	2.10 (s)	COMe-3	1.90 (s)	1.95 (s)
COMe-5	2.08 (s)	COMe-5	-	-
COMe-7	2.19 (s)	COMe-7	-	-
COMe-9	2.01 (s)	COMe-9	-	-
COMe-15	1.96 (s)	COMe-15	2.32 (s)	-
Benzoyls	-	Benzoyls	-	-
COPh-8 -2,6	8.05 (m)	COPh-5-2,6	7.56 (m)	7.48 (m)
-3,5	7.44 (m)	-3,5	7.03 (m)	6.88 (m)
-4	7.55 (m)	-4	7.22 (m)	7.06 (m)
COPh-7-2,6	-	COPh-7-2,6	7.51 (m)	7.53 (m)
-3,5	-	-3,5	6.93 (m)	7.02 (m)
-4	-	-4	7.10 (m)	7.25 (m)
OH-1	-	OH-1	3.94 (brs)	-
OH-2	-	OH-2	2.53 (m)	2.33 (s)
OH-8	-	OH-8	3.59 (m)	3.50 (m)
OH-15	-	OH-15	-	4.10 (s)

**Table 2:** The ^1^H-NMR spectral assignments (δ/ppm) for compounds **4**–**9** in CDCl_3_ (300 MHz).

Position	4	5	6	7	8	9
1	6.14 (brs)	6.05 (s)	6.04 (s)	6.07 (s)	6.14 (s)	6.02 (s)
3	5.07 (s)	5.60 (s)	5.57 (s)	5.53 (s)	5.67 (s)	3.73 (s)
5	5.51 (brs)	3.88 (s)	3.88 (s)	3.68 (s)	3.76 (brs)	5.41 (s)
7	5.88 (d, 1.5)	6.12 (d, 3.7)	6.12 (d, 3.7)	5.76 (m)	5.78 (m)	5.91 (m)
8	4.26 (dd, 9.5, 1.5)	4.08 (dd, 4.0, 11.7)	4.08 (dd, 4.0, 11.7)	4.02 (dd, 9.9, 3.0)	4.03 (m)	4.13 (dd, 7.0, 14.2)
11	2.31 (m)	2.49 (m)	2.48 (m)	2.46 (m)	2.54 (m)	2.41 (m)
12	2.54 (m)	2.26 (m)	2.26 (m)	2.26 (m)	2.28 (m)	2.34 (m) 1.77
	1.73 (m)	1.75 (m)	1.76 (m)	1.74 (m)	1.75 (m)	(m)
13	0.74 (m)	0.72 (m)	0.72 (m)	0.67 (m)	0.67 (m)	0.70 (m)
14	0.88 (m)	0.98 (m)	0.98 (m)	0.90 (m)	0.93 (m)	0.94 (m)
16	1.07 (s)	1.06 (s)	1.06 (s)	1.05 (s)	1.04 (s)	1.07 (s)
17	1.13 (s)	1.08 (s)	1.08 (s)	1.08 (s)	1.06 (s)	1.17 (s)
18	1.00 (d, 4.7)	1.00 (d, 7.3)	1.00 (d, 7.3)	0.99 (d, 7.2)	1.05 (d, 4.2)	0.98 (d, 5.61)
19	1.78 (brs)	1.8 (d, 1.1)	1.8 d (1.1)	1.80 (brs)	1.83 (d, 1.5)	1.83 (s)
20	1.56 (s)	4.75, 4.49 (Abq, 12.5)	4.75, 4.49 (Abq, 12.5)	1.79 (s)	1.81 (s)	1.60 (s)
3-R, 2′	8.13 (m)	5.95 (d, 15.3)	5.86 (d, 15.3)	5.95 (d, 5.1)	8.04 (d, 7.3)	-
3′	7.48 (m)	7.70 (m)	7.31 (m)	7.68 (dd, 15.3, 11.4)	7.48 (t, 7.6)	-
4′	7.60 (m)	6.16 (m)	6.21 (m)	6.16 (m)	7.61 (t, 7.3)	-
5′	-	5.95 (m)	6.21 (m)	5.94 (m)	-	-
6′	-	2.29 (m)	2.20 (m)	2.34 (m)	-	-
7′	-	1.43 (m)	1.43 (m)	1.43 (m)	-	-
8′	-	1.31 (m)	1.31 (m)	1.29 (m)	-	-
9′	-	1.28 (m)	1.28 (m)	-	-	
10′	-	0.89 (t, 7.0)	0.72 (t, 7.0)	0.89 (m)	-	-
OAc	-	2.06 (s)	2.05 (s)	-	-	-
5-R, 2′	2.05 (s)	-	-	-	-	8.10 (m)
3′	-	-	-	-	-	7.46 (m)
4′	-	-	-	-	-	7.60 (m)

**Table 3:** The ^1^H-NMR spectral assignments (δ/ppm) for compounds **10** and **11** in CDCl_3_ (300 MHz).

Position	10	11
1α	1.44 (m)	1.44 (m)
1β	1.86 (m)	1.86 (m)
2	1.76 (m)	1.75 (m)
	1.67 (m)	1.66 (m)
3α	3.29 (dd, 4.6,11.6)	3.29 (dd, 4.6,11.6)
5α	1.67 (m)	1.66 (m)
6α	2.40 (m)	2.41 (m)
6β	2.38 (m)	2.38 (m)
11α	2.37 (m)	2.36 (m)
11β	2.24 (m)	2.24 (m)
12α	1.76–1.80 (m)	1.76–1.80 (m)
15α	1.55 (m)	1.55 (m)
15β	2.12 (m)	2.12 (m)
16α	1.34 (m)	1.34 (m)
16β	1.93 (m)	1.94 (m)
17	1.44 (m)	1.46 (m)
18	0.73 (s)	0.73 (s)
19	1.06 (s)	1.05 (s)
20	1.41 (m)	1.43 (m)
21	0.88 (d, 6.0)	0.93 (d, 6.1)
22	1.13 (m), 1.55 (m)	1.05 (m), 1.48 (m)
23	1.90 (m), 2.04 (m)	1.82 (m), 2.04 (m)
24	5.09 (m)	5.10 (m)
26	1.69 (s)	1.68 (s)
27	1.61 (s)	1.60 (s)
28	0.99 (s)	0.99 (s)
29	0.88 (s)	0.88 (s)
30	0.97 (s)	0.96 (s)

**Table 4:** The proliferation inhibition activities of kansui extract and compounds **1**–**12** on human normal liver cells L-O2 and gastric epithelial cell GES-1 (*n* = 6).

Extract/Compounds	IC_50_	

	L-O2	GES-1
EtOH extract	42.02 μg/mL	30.67 μg/mL
EtOAc extract	27.08 μg/mL	21.89 μg/mL
Water extract	>100 μg/mL	>100 μg/mL
1	>100 μM	>100 μM
2	>100 μM	>100 μM
3	>100 μM	>100 μM
4	>100 μM	>100 μM
5	12.40 μM	8.51 μM
6	8.22 μM	6.67 μM
7	13.08 μM	3.53 μM
8	70.78 μM	23.51 μM
9	34.53 μM	22.01 μM
10	14.36 μM	13.44 μM
11	17.82 μM	8.04 μM
12	>100 μM	>100 μM
